# Biocompatible and Hermetic Encapsulation of PMUTs: Effects of Parylene F-VT4 and ALD Stacks on Membrane Vibration and Acoustic Performance

**DOI:** 10.3390/s25134074

**Published:** 2025-06-30

**Authors:** Esmaeil Afshari, Samer Houri, Rik Verplancke, Veronique Rochus, Maarten Cauwe, Pieter Gijsenbergh, Maaike Op de Beeck

**Affiliations:** 1 IMEC—Interuniversity Microelectronics Centre, Kapeldreef 75, 3001 Leuven, Belgium; 2 Centre for Microsystems Technology (CMST), Technologiepark-Zwijnaarde 126, 9052 Ghent, Belgium

**Keywords:** PMUT, hermetic encapsulation, flexible coating, implantable ultrasonic devices

## Abstract

The motivation of this work is to enable the use of piezoelectric micromachined ultrasonic transducer (PMUT)-based implants within the human body for biomedical applications, particularly for power and data transfer for implanted medical devices. To protect surrounding tissue and ensure PMUT functionality over time, biocompatible and hermetic encapsulation is essential. This study investigates the impact of Parylene F-VT4 layers of various thicknesses as well as the effect of multilayer stacks of Parylene F-VT4 combined with atomic layer-deposited nanolayers of Al_2_O_3_ and HfO_2_ on the mechanical and acoustic properties of PMUTs. PMUTs with various diameters (40 µm, 60 µm, and 80 µm) are fabricated and tested both as stand-alone devices and as arrays. The mechanical behavior of single stand-alone PMUT devices is characterized in air and in water using laser Doppler vibrometry (LDV), while the acoustic output of arrays is evaluated by pressure measurements in water. Experimental results reveal a non-monotonic change in resonance frequency as a function of increasing encapsulation thickness due to the competing effects of added mass and increased stiffness. The performance of PMUT arrays is clearly influenced by the encapsulation. For certain array designs, the encapsulation significantly improved the arrays’ pressure output, a change that is attributed to the change in the acoustic wavelength and inter-element coupling. These findings highlight the impact of encapsulation in modifying and potentially enhancing PMUT performance.

## 1. Introduction

As the field of biomedical implants continues to advance, the demand for effective means of wireless transmission becomes increasingly vital for achieving power and data transfer with the implants [1,2]. Traditionally, most implantable devices rely on batteries for power, which can lead to bulky devices and require regular battery replacement in the case of long-term implantation, thus requiring invasive medical intervention. Additionally, implantable devices usually require communication capabilities to interact with external wearable devices or controllers placed outside the human body [3,4]. Such communication capability is essential for various purposes, including device parameter adjustment (e.g., stimulation parameters for a pacemaker or deep brain stimulator), transmission of stored information (e.g., stored electro-cardiogram), and real-time pain management (e.g., dynamic stimulation adjustments between neurostimulators) [5,6,7].

Both the connection to the outside world and the power supply for implanted devices can be provided by wireless technology while avoiding the issues associated with wired connection, with the most important issues being the severe risk of infection and reduced patient comfort since a connected wearable device has to be worn continuously [8,9]. While several wireless technologies can be utilized for data and power transfer for implantable medical devices, ultrasound (acoustic) waves offer a unique set of advantages, particularly for deep implants. Optical and infrared radiation methods are not suitable for deep tissue applications due to significant scattering and absorption in biological tissues [10,11]. RF-based electromagnetic waves, on the other hand, face challenges for deep implantation due to significant power absorption by local tissue at legally allowed frequencies and powers, resulting in very poor power transmission and tissue heating [3]. In contrast, ultrasound waves propagate efficiently through the soft tissue with very limited power absorption, making them well-suited for deep implants [12]. Ultrasonic waves also provide an advantage in terms of data security since they are only captured in the immediate vicinity of the human body due to the large acoustic impedance mismatch between tissue and air [13]. Recent work highlighted the effectiveness of ultrasound-based technologies in enabling both communication and wireless power transfer for implantable biomedical devices, including PMUT-based solutions designed for brain implants and charging applications [14,15,16].

Most commonly, ultrasound waves are generated by bulk piezoelectric transducers; however, their size, the difficulty of integration, and lack of design flexibility limit their use for implantable devices [17]. These limitations can be overcome by employing a micromachined ultrasonic transducer (MUT), which offers the advantage of a small footprint and the possibility of integrated electronics. MUTs also provide an inherently wide bandwidth [18], higher frequency of operation [19], low self-heating [20], and a large amount of freedom in designing the transducer geometry and its footprint compared to bulk ultrasonic transducers [21].

MUTs can be classified into two types based on their transduction mechanisms: capacitive micromachined ultrasonic transducers (CMUTs), which rely on flexural vibrations induced by the electrostatic attraction between a suspended membrane and the substrate [22,23], and piezoelectric micromachined ultrasonic transducers (PMUTs), which generate flexural vibrations in the suspended membrane via the piezoelectric effect [24,25]. In PMUTs, out-of-plane membrane deflection is driven by the lateral strain generated within the piezoelectric material, which means membranes must include at least a piezoelectric layer, such as aluminum nitride (AlN) or lead zirconate titanate (PZT). An additional important design consideration for PMUTs is the inclusion of an elastic layer. The elastic layer in a PMUT is crucial to transforming in-plane stresses into out-of-plane deformation; therefore, its thickness should be optimized. Optimizing the elastic layer’s thickness and material properties ensures the right balance between stiffness and sensitivity, maximizing the PMUT’s performance in its intended application. Recent reviews and simulation studies highlight the growing focus on PMUT technologies for biomedical applications, particularly emphasizing the modeling and design of large PMUT arrays for medical use [22,26].

When used for medical purposes inside the body, an effective encapsulation is essential for MUTs to electrically, chemically, and physiologically insulate the device from the outside environment: the device should be protected against humidity and other corrosive elements in the body, and biocompatibility should be ensured [27,28]. Conventional rigid implant encapsulation materials such as titanium, titanium alloys, and glass, provide excellent barrier properties, but are generally incompatible with MUTs due to their stiffness. This rigidity hinders the motion of the MUT membrane and reduces vibration amplitudes, all diminishing the acoustic output. In contrast, biocompatible polymers such as polyimide, Parylene, and polydimethylsiloxane (PDMS) are potential candidates for encapsulation of biomedical implantable devices since these polymers exhibit properties such as flexibility, biocompatibility, and hermeticity [29]. While these biocompatible polymers on their own already have very good barrier properties, multilayer encapsulations of these polymers combined with ceramic nanolayers fabricated by atomic layer deposition (ALD) can result in extremely good barrier properties, enabling ultra-long term device functionality in the human body [30].

Among the various Parylene types, Parylene F-VT4 is a particularly interesting candidate for encapsulation of mechanical implantable devices due to its mechanical flexibility, chemical resistance, and biocompatibility [31,32]. Parylene F-VT4 also offers greater thermal stability compared to other types of Parylene, making it compatible with thermal ALD processes. ALD allows for the very accurate deposition of extremely thin and uniform ceramic nanolayers, which, when combined with polymers, create a highly effective encapsulation layer. Li et al. [33] developed a sandwiched stack of ALD layers (HfO_2_/Al_2_O_3_/HfO_2_) and proved that the combination of this ALD stack and polyimide layers improves the barrier properties and the lifetime of the encapsulation, providing a very robust protection for the implantable devices.

Few systematic studies exist regarding the impact of encapsulation on MUTs’ behavior. Depending on the encapsulation layer thickness and its material properties, encapsulation may significantly alter the dynamic behavior of the MUT membrane, potentially enhancing or hindering its performance [13,34,35,36,37]. Thus, an in-depth investigation of the influence of encapsulation on key performance parameters such as resonance frequency, displacement, and acoustic output is still required for achieving improved device functionality and optimal device designs.

The purpose of this study is to systematically investigate the effects of encapsulation layers—specifically, Parylene F-VT4 with varying thicknesses and encapsulation stacks consisting of Parylene F-VT4 combined with ALD-deposited ceramic nanolayers—on the mechanical and acoustic properties of PMUTs in view of their potential for implantable biomedical devices. In addition to the introduction, this paper is divided into four sections. Section 2 describes the fabricated devices and the experimental setups and procedures employed in this work. Section 3 delves into the modeling of the mechanical effects of encapsulation on the PMUT properties. In Section 4, the measurement results are presented and discussed in detail, while Section 5 provides the final summary and conclusion. By providing insights into the dynamic interactions between encapsulation thickness, material properties, and PMUT behavior, this paper aims to advance the development of PMUT encapsulation strategies for reliable, high-performance ultrasound devices for biomedical applications.

## 2. Devices and Methods

### 2.1. PMUT Description

**Figure 1:**
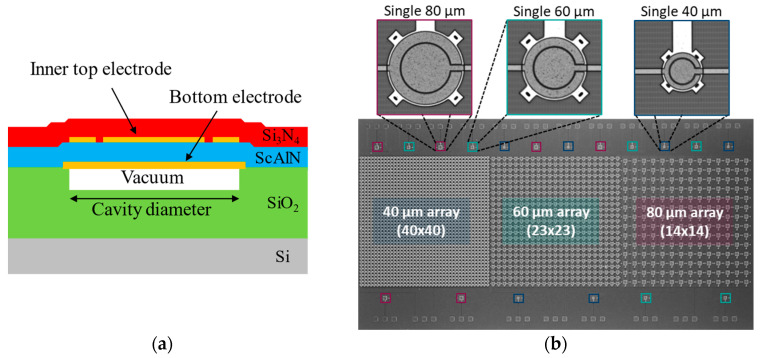
(**a**) Cross section of a single PMUT (MEMS only); (**b**) optical image of the fabricated PMUT chip.

The cross-sectional diagram of the investigated PMUTs is indicated in Figure 1a, and these devices were fabricated by SilTerra Malaysia in Kulim, Malaysia, using a foundry-compatible MEMS process [38,39]. These PMUTs consist of a 1.3 μm-thick 9.5% scandium-doped aluminum nitride (AlSc9.5N) piezoelectric layer, which is sandwiched between two aluminum electrodes; the bottom electrode has a thickness of 400 nm, while the top electrode is 350 nm thick. In alignment with prior research finding, the ratio of the inner top electrode diameter (D’) to the bottom electrode diameter (D) was optimized at D′/D = 67%, aiming for a first mode maximal driving efficiency [40,41]. A 1.5 μm silicon nitride layer is deposited on top of the metal-sandwiched piezoelectric stack as an elastic layer. To form the circular PMUT structure, a 0.6 μm deep circular cavity is etched into the underlying silicon dioxide layer, which is 4 μm thick and sits on a silicon substrate.

To evaluate the impact of encapsulation layers on the mechanical behavior of PMUTs, single PMUT devices with three different cavity diameters—40 µm, 60 µm, and 80 µm—are fabricated and measured. These varied diameters provide insights into the dependence of the encapsulated PMUT properties on diameter. In addition, PMUT arrays were also equally fabricated to explore how encapsulation layers influence the pressure output of PMUTs arranged in arrays. Each array, despite having different individual PMUT cavity diameters, is designed to occupy a similar total active area of approximately 2.8 mm×2.8 mm. Due to the varying sizes of the individual PMUTs, each array comprises a different number of devices: thus, the 40 µm, 60 µm, and 80 µm device arrays contain 40 × 40, 23 × 23, and 14 × 14 devices, respectively. An optical image of the PMUT chip, including the single PMUTs and PMUT arrays, is shown in Figure 1b.

In each array, all inner top electrodes of the elements are connected together, as are all bottom electrodes, allowing for collective actuation. Wafers containing the PMUT arrays are diced into chips, with each chip containing the three arrays along with stand-alone devices. These chips are mounted on a custom-designed printed circuit board (PCB) and wire-bonded to allow electrical actuation. Following this, the wire bonds were covered with epoxy to protect them from mechanical damage and electrical shorting since they need to be tested in a fluidic environment.

### 2.2. Encapsulation Layer Deposition

**Figure 2:**
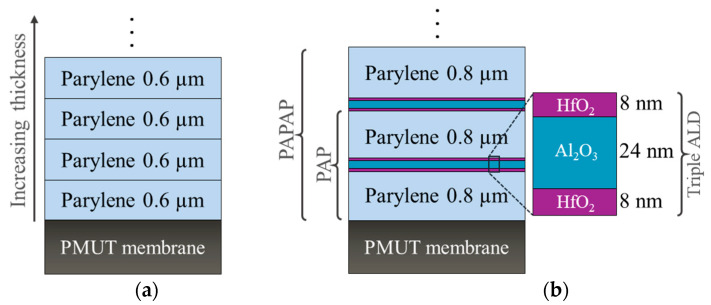
Schematic illustration of encapsulation layers; (**a**) only Parylene F-VT4 of different thicknesses, (**b**) multilayer encapsulation stacks (PAP: 0.8 µm Parylene/triple ALD/0.8 µm Parylene).

As previously stated, two sets of encapsulation stacks are used, one consisting of Parylene F-VT4 layers only, while the second stack consists of alternating layers of Parylene F-VT4 and ALD-deposited ceramic layers. For clarity, “Parylene” will be used throughout the rest of the paper to refer to Parylene F-VT4. The Parylene layers were deposited on the PMUT devices using a chemical vapor deposition (CVD) process, utilizing the C30S Comelec equipment at room temperature. Prior to Parylene deposition, samples are treated with silane A174 as an adhesion promoter. This CVD method enables uniform and conformal deposition, ensuring the encapsulation layer adheres effectively to the PMUT surface without introducing defects [42,43]. To investigate the influence of various Parylene thicknesses on the PMUT’s mechanical and acoustic performance, Parylene was coated up to a thickness of 10.2 µm in increments of 0.6 µm, as illustrated in Figure 2a.

In the case of the multilayer encapsulation stacks consisting of alternating layers of Parylene and atomic layer deposition (ALD) films, the process began with a 0.8 µm Parylene layer, followed by the deposition of a triple-layer ALD stack consisting of 8 nm of hafnium oxide (HfO_2_), 24 nm of aluminum oxide (Al_2_O_3_), and 8 nm of hafnium oxide (HfO_2_). These films were deposited in a single thermal ALD process at 120 °C using a Savannah ALD system (Veeco). ALD is a vapor-phase thin-film deposition technique based on alternating, self-limiting surface reactions, also called ‘half-reactions’. In each cycle, the substrate is first exposed to a metal-containing precursor, which chemisorbs onto the surface. This first half-reaction is a self-limiting reaction, resulting in the coverage of the surface with a metal-containing monolayer. A nitrogen purge will remove the remaining metal-containing precursor, and the surface is subsequently exposed to a co-reactant (typically an oxidant), forming a monolayer of the desired material (i.e., Al_2_O_3_) during the second half-reaction. This cycle of two half-reactions is repeated to create monolayer after monolayer on the surface with atomic-scale precision in order to build up the desired thin film with highly accurate thickness and layer composition with an almost perfect step coverage over topography. Trimethylaluminum and water are used as precursors for the aluminum oxide deposition. For hafnium oxide deposition, tetrakis(dimethylamido)hafnium and water are used as precursors. The Parylene and ALD layers are deposited in an alternating way, adding 0.8 µm Parylene after each ALD stack, creating a multilayer structure. For convenience, the stack configuration of Parylene/ALD-3/Parylene will be denoted as PAP, where “P” refers to the Parylene layer, and “A” represents the ALD stacks as shown in Figure 2b. Up to five repetitions of Parylene-ALD layers (“PAPAPAPAPAP”) were evaluated.

In this work, three identical chip samples were always used to investigate each thickness of Parylene or encapsulation stacks in order to enhance the accuracy of the investigation. As shown in Figure 1b, each chip contains six single PMUTs of each cavity diameter, allowing the evaluation of each encapsulation on 18 single PMUTs, along with three arrays (an array for each diameter). All samples are characterized before and after encapsulation in air as well as in water.

### 2.3. Vibration Measurement

**Figure 3:**
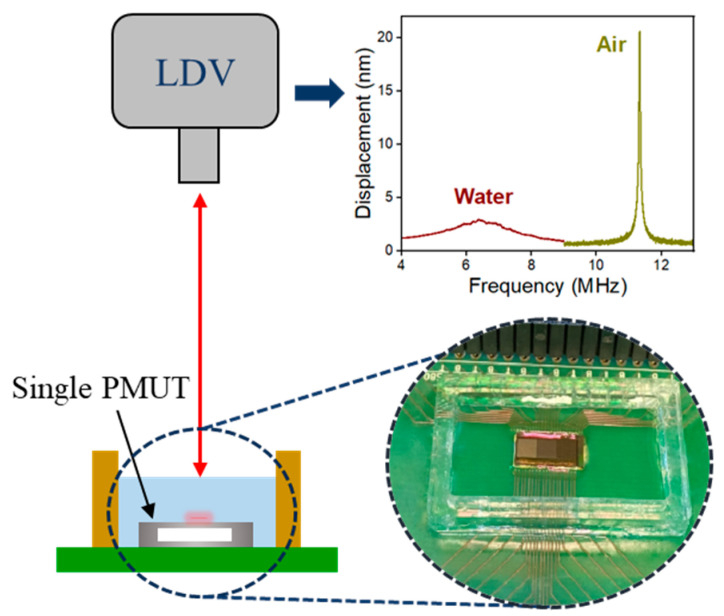
Laser Doppler vibrometry measurement setup, and an example of in-air and in-water frequency sweeps.

A Polytec MSA-500 laser Doppler vibrometer (LDV), manufactured by Polytec in Waldbronn, Germany, is used to study the impact of encapsulation on the resonance frequencies, vibration amplitudes, and quality factors of PMUTs, in air and in water. The frequency response of each PMUT was determined by applying a frequency sweep to the inner top electrode through a Keysight 33500B arbitrary waveform generator (AWG) from Santa Rosa, CA, USA, while grounding the bottom electrode and leaving the outer electrode floating. Once the resonance frequency is identified, a continuous sinusoidal signal of 1V_pp_ at this particular frequency is applied, and the displacement at the center of the membrane is measured. For the in-water vibration measurements, a fluid-filled container is constructed by attaching a PMMA ring onto the PCB upon which the PMUT chip is wire-bonded, this container is filled with high-purity water, thus immersing the PMUTs, as shown in Figure 3.

### 2.4. Acoustic Measurement

**Figure 4:**
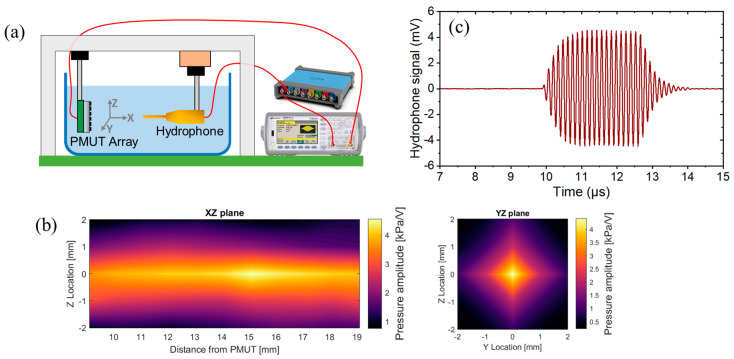
(**a**) Experimental setup of transmission measurement in water, (**b**) example of a 3D scan of the focal point for an uncoated 60 µm PMUT array at its peak frequency, and (**c**) hydrophone signal at the focal point for the uncoated 60 µm PMUT array at its peak frequency.

The acoustic performances of uncoated and coated PMUT arrays, both in transmission and receive modes, are conducted in a water tank maintained at room temperature. For transmission characterization, a Keysight 33500B AWG is used to produce a sinusoidal voltage signal with an amplitude of 10 Vpp (burst count 20) in order to actuate the PMUT elements. A commercial 0.5 mm needle hydrophone (Precision Acoustics, Dorchester, UK) is securely positioned facing the PMUT array and connected to a digital oscilloscope (Teledyne LeCroy HDO4024A, Chestnut Ridge, NY, USA) to record the received voltage signal, as shown in Figure 4a. The received signal recorded by the hydrophone will be termed the “hydrophone signal” further in this text.

To determine the peak frequency, the distance between the PMUT chip and the hydrophone is initially set in the far field region for each array. For this reason, the focal distance is roughly estimated using the Rayleigh equation, yielding approximate values of 32 mm, 15 mm, and 9 mm for the 40 µm, 60 µm, and 80 µm arrays, respectively. Based on these estimates, fixed distances of 40 mm, 30 mm, and 20 mm are chosen for arrays of 40 µm, 60 µm, and 80 µm, respectively. At each fixed distance, a 2D scan is conducted to locate the position of the maximum pressure point for each array. Once this maximum point is identified, a frequency sweep is applied to precisely measure the peak frequency.

After determining the peak frequency, the PMUT arrays were actuated at these peak frequencies, and a 3D scan is performed to measure the focal point accurately. An example of this 3D scan is presented in Figure 4b for an uncoated array of 60 µm-diameter PMUTs. Finally, the hydrophone signal at the peak frequency is measured at the focal point. Figure 4c shows the hydrophone signal at the focal point for the uncoated 60 µm array. The hydrophone signal is then converted into pressure using the hydrophone’s sensitivity.

**Figure 5:**
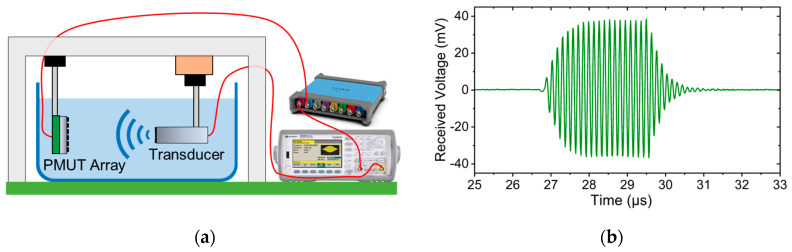
(**a**) Experimental setup in the receive mode; (**b**) received signal recorded by an uncoated 60 µm array in the receive mode.

The sensitivity of the PMUT device as a receiver is characterized by using an Olympus V321-SU transducer (manufactured by Olympus Corporation, Waltham, MA, USA) as an ultrasonic source. First, the pressure output of the transducer at different frequencies is measured using the needle hydrophone at a fixed distance of 4 cm. The transducer has a center frequency of 10 MHz and a −6 dB bandwidth of approximately 12.56 MHz (120%). The pressure of the transducer at 10 MHz is measured at 85 kPa for an applied voltage of 10 Vpp. For sensitivity measurement, as shown in Figure 5a, the PMUT arrays are immersed in water and aligned facing the transducer at 4 cm. The received signals from the PMUT arrays were captured using the digital oscilloscope. Figure 5b shows an example of a received voltage when an uncoated 60 µm array is used as a receiver. The sensitivity of the PMUT arrays is determined by dividing the received signal amplitude by the pressure output of the Olympus transducer.

## 3. Modeling and Numerical Simulation

We developed an analytical model for our encapsulated PMUTs based on established MEMS and PMUT modeling approaches. This approach provides a high-level understanding of how various parameters influence the performance of the PMUT arrays. The analytical framework provides fundamental insights into device behavior, facilitating a more informed design process before engaging in numerical simulations or experimental validation.

### 3.1. Resonance Frequency of a Circular Multilayered Plate

**Figure 6:**
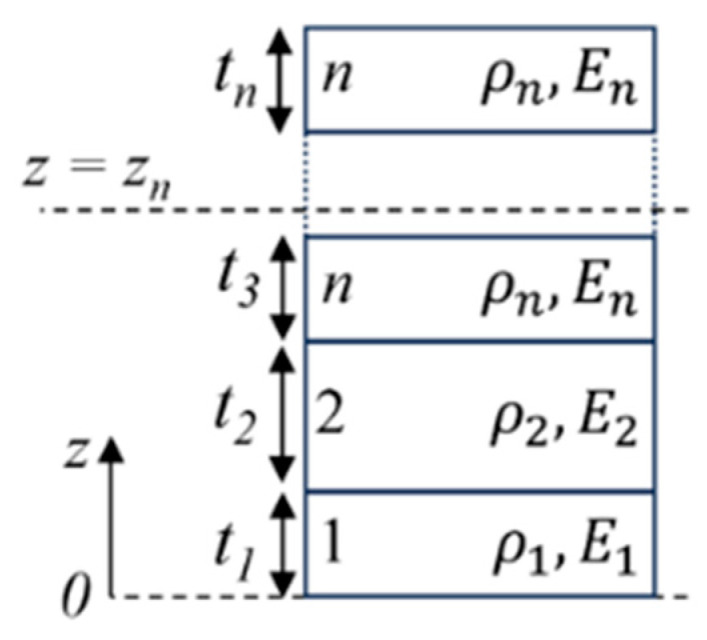
Schematic of a multilayered plate with *n* layers and neutral axis *Z_n_*.

To analytically model the impact of encapsulations on the resonance frequency of the PMUT, the structure is approximated as a multilayered stress-free uniform plate. This approach allows for a simplified yet effective analysis of how varying encapsulation layers influence the resonance frequency. The resonance frequency of the fundamental mode $f_{r}$ for a multilayered circular plate can be described by the following equation [44]:

(1) $$f_{r}=\frac{1.6259}{a^{2}}\;\sqrt{\;\frac{D_{eq}}{I_{0\;eq}}}$$

where a is the radius of the plate, $D_{eq}$ represents the equivalent flexural rigidity of the plate, and $I_{0\;eq}$ is the equivalent areal mass density of the plate. For a circular plate with n layers, as illustrated in Figure 6, where each layer has a thickness $t_{i}$, density $\rho_{i}$, Young’s modulus $E_{i}$, and Poisson’s ratio $v_{i}$, the expressions for $D_{eq}$ and $I_{0\;eq}$ are given by the following equations [45]:

(2) $$D_{eq}=\frac{1}{3}\overset{n}{\underset{i=1}{\sum }}\frac{E_{i}}{1-v_{i}^{2}}\left[{\left(\overset{i}{\underset{j=1}{\sum }}t_{i}-z_{n}\right)}^{3}+{\left(z_{n}-\overset{i-1}{\underset{j=1}{\sum }}t_{j}\right)}^{3}\right]$$

(3) $$I_{0\;eq}=\overset{n}{\underset{i=1}{\sum }}\rho_{i}t_{i}.$$

Here, $z_{n}$ is the neutral axis where the bending strain is zero, and it depends on the geometry and stiffness of each material layer. Assuming Poisson’s ratio of each layer $v_{i}$ is similar, the neutral plane $z_{n}$ is defined as follows [46]:

(4) $$z_{n}=\frac{\sum_{i=1}^{n}E_{i}t_{i}\left(\sum_{j=1}^{i-1}t_{j}+\frac{1}{2}t_{i}\right)}{\sum_{i=1}^{n}E_{i}t_{i}}.$$

### 3.2. Fluid Loading Effect on the Resonance Frequency

When the PMUT operates in a fluid medium, the surrounding fluid introduces additional resistance and mass to the vibrating membrane. This added mass increases the total inertia of the membrane, which results in a reduction in the resonance frequency $f_{f}$, as given by [47,48]:

(5) $$f_{f}=\frac{1}{\sqrt{1+\beta }}f_{r}.$$

Here, β is the added virtual mass incremental factor (AVMI factor), a dimensionless quantity that depends on properties of the fluid and the membrane size, expressed as follows:

(6) $$\beta =\frac{\Gamma \rho_{f}a}{I_{0}}$$

where Γ is called the non-dimensionalized added virtual mass incremental factor (NAVMI factor), $\rho_{f}$ is the density of the fluid, a is the plate radius, and $I_{0}$ is the areal mass density of the circular plate. There exists some debate regarding the most accurate approximation for Γ, with researchers employing different approaches under distinct boundary conditions (such as clamped, free edge, and simply supported) and their analytical calculation estimate Γ to range from 0.4945 to 1.045 [48]. A rough estimate for Γ can be derived by comparing the resonance frequency of PMUTs in water $f_{w}$ and air $f_{a}$, assuming that the influence of air on the resonance frequency is negligible. Under this assumption, Equations (5) and (6) yield the following equation:

(7) $${\left(\frac{f_{a}}{f_{w}}\right)}^{2}-1=\frac{\Gamma \rho_{w}a}{I_{0}}$$

where $\rho_{w}$ represents the density of water, a is the plate radius, and $I_{0}$ is the areal mass density. A plot of ${\left(\frac{f_{a}}{f_{w}}\right)}^{2}-1$ versus $\frac{\rho_{w}a}{I_{0}}$ will yield a linear relationship, where the slope corresponds to the value of Γ.

### 3.3. Effect of Encapsulation on Resonance Frequency

To investigate the impact of encapsulation layers on the PMUT resonance frequency, an analytical calculation is conducted using the analytical models outlined above for the resonance frequency of a multilayered plate. The material properties used here are listed in Table 1.

**Figure 7:**
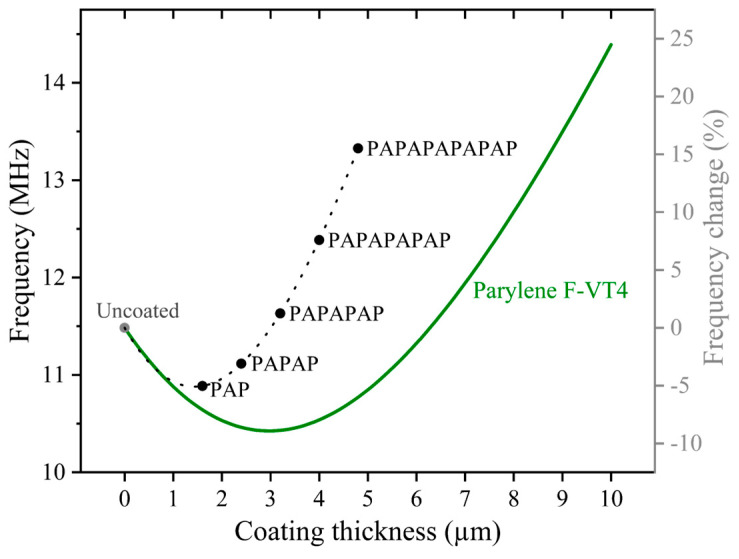
Analytical results of the resonance frequency for a 60 µm PMUT membrane covered with varying Parylene F-VT4 thicknesses (solid line) and with stacks of Parylene F-VT4 combined with ALD (represented by dots). The dashed line added to the results of the encapsulation stack is a guide for the eye.

Figure 7 shows the results of this calculation for a plate with 60 µm diameter for different thicknesses of Parylene and encapsulation stacks. The resonance frequency exhibits a non-monotonic behavior as the thickness of Parylene increases, which is attributed to the combined effects of added mass and altered stiffness due to encapsulation. The added mass generally lowers the resonance frequency, while the increased stiffness tends to raise it. As Parylene thickness increases, the added mass effect dominates initially and thus decreases the resonance frequency. Above ~3 µm Parylene, the added stiffness effect dominates and increases the resonance frequency. In case of encapsulation stacks, the observed resonance frequencies are higher compared to those of only Parylene (without ALD layers). This is related to the significantly higher Young’s modulus of ALD layers compared to Parylene, which leads to a substantial increase in the equivalent flexural rigidity of the Parylene/ALD stacks, and, consequently, a higher resonance frequency.

### 3.4. Effect of Encapsulation on Displacement

Adding encapsulation layers on a membrane and increasing its thickness changes the membrane’s displacement. The change in displacement can be attributed to several factors: change in dissipation and change in transduction efficiency (the efficiency of transforming an electrical signal into a mechanical displacement). The first and main factor is the dissipation introduced by the encapsulation layer. As the thickness of the polymer-based encapsulation layer increases, the energy lost through added viscous damping increases. The increased energy loss reduces the amplitude of oscillation, leading to a decrease in the displacement of the membrane’s center.

The second factor affecting the displacement is the change in the transduction efficiency, which results from the interplay of the shift in the neutral axis and the change in stiffness due to the addition of encapsulation layers. In the literature, a figure of merit for the transduction efficiency known as the “effective electromechanical coupling coefficient” ($k_{eff}^{2}$) is used to indicate the efficiency of transforming the electrical energy into the mechanical domain [55]. This $k_{eff}^{2}$ is usually measured via electrical one-port impedance measurements. In this work, due to the difficulties in accurate one-port electrical measurements, we define the transduction efficiency to be the ratio of displacement to input voltage, and thus in units of nm/V.

The origin of the change in the transduction coefficient can be attributed to the fact that the overall bending force is directly influenced by the position of the neutral axis; as the neutral axis shifts due to the addition of encapsulation layers, the distance between the piezoelectric layer and the neutral axis increases, resulting in a higher bending force. Simultaneously, the stiffness of the membrane increases with the encapsulation layers’ thickness, making the entire structure more rigid. This leads to a non-monotonic variation in transduction efficiency, which reaches a peak at a specific thickness of the encapsulation layer. We define the transduction efficiency mathematically as follows:

(8) $$Transduction\;efficiency=\frac{F_{b}}{k}$$

where $F_{b}$ is the bending force acting on the membrane, which is considered proportional to the distance of the neutral axis from the center of the piezoelectric layer, i.e., $z_{n}-z_{c}$, while k is the stiffness, which is proportional to the equivalent flexural rigidity $D_{eq}$ [56]. Thus, we define the transduction factor as the ratio of this distance $z_{n}-z_{c}$ to the equivalent flexural rigidity $D_{eq}$:

(9) $$Transduction\;factor\propto \;\frac{z_{n}-z_{c}}{D_{eq}}$$

**Figure 8:**
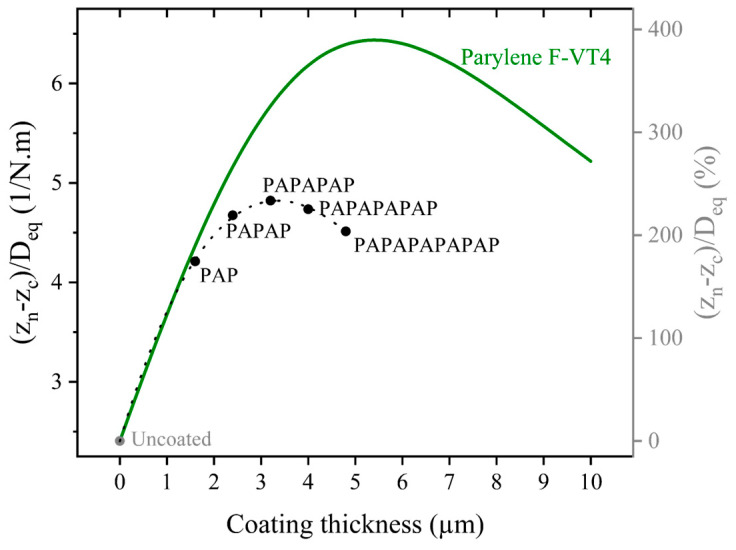
Analytical results of $\left(\left(z_{n}-z_{c}\right)/D_{eq}\right)$ as a function of Parylene thickness (solid line) and encapsulation stacks of Parylene and ALD (dots). The dashed line added to the encapsulation stack results is a guide for the eye.

Figure 8 illustrates the change in analytical transduction factor, which is the ratio of the distance to the analytically calculated neutral axis (from Equation (4)) to the equivalent flexural rigidity (from Equation (2)) for different thicknesses of Parylene and encapsulation stacks.

Transduction efficiency can also be described in terms of the membrane’s displacement and quality factor. According to Hooke’s law, the static displacement is proportional to the applied force divided by the stiffness of the membrane. The dynamic displacement (*D_d_*), on the other hand, is given by the product of the quality factor (*Q*) and the static displacement (*D_s_*). This relationship holds under the assumption that the static and dynamic stiffness of the structure are equivalent. However, even if this condition is not strictly met, the equation remains valid when considering only the relative change at resonance. This relationship can be expressed as follows:

(10) $$D_{d}=Q\;\cdot \;D_{s}=Q\;\cdot \;\frac{F_{b}}{k}$$

From this, we can also express the transduction efficiency in terms of the dynamic displacement and quality factor:

(11) $$Transduction\;efficiency=\frac{F_{b}}{k}=\frac{D_{d}}{Q}$$

In the results, the measured transduction efficiency is derived from the experimentally measured displacement and quality factor for PMUTs based on the above equation.

### 3.5. FEM Simulation Using COMSOL

**Figure 9:**
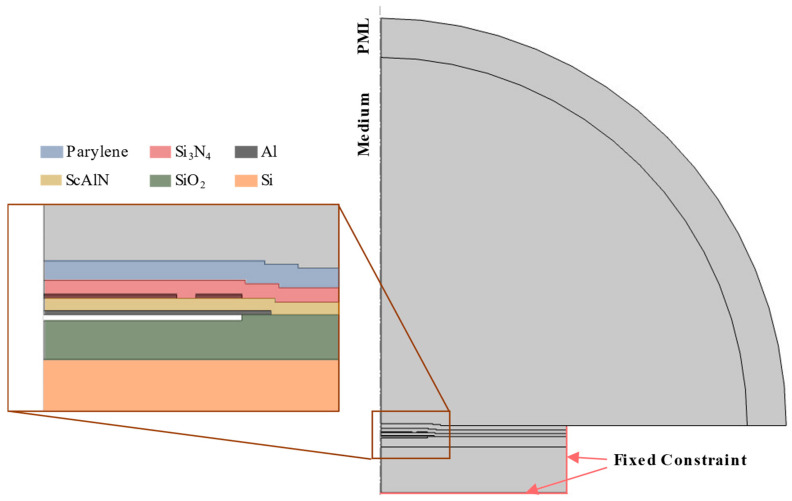
Illustration of 2D axisymmetric model of the PMUT encapsulation setup in COMSOL Multiphysics, for the case of a single Parylene layer as encapsulation.

A 2D axisymmetric model was created in COMSOL Multiphysics version 6.1 to simulate the influence of encapsulation on the mechanical behavior of the PMUT’s membrane (Figure 9). The geometry consists of the PMUT membrane stack and the encapsulation layers as described in previous sections. The material properties used in the simulation are identical to those listed in Table 1, with the properties for SiO_2_ and silicon sourced from the COMSOL material library. Simulations were performed using the Solid Mechanics, Electrostatics, and Pressure Acoustics modules. An acoustic domain, consisting of air or water, is added above the PMUT and surrounded by a perfectly matched layer (PML) to absorb acoustic pressure and minimize reflections that simulate an infinite domain. A harmonic voltage perturbation is applied to the top electrode, while the bottom electrode is grounded. Resonance frequencies are extracted from the center of the PMUT diaphragm using a frequency domain simulation. The results of these simulations are shown in Section 4.

## 4. Results and Discussion

**Figure 10:**
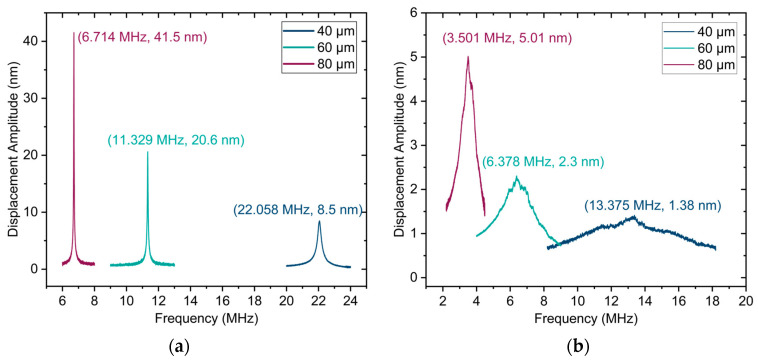
Mechanical performance of uncoated single PMUTs of various dimensions: (**a**) in air; (**b**) in water.

The mechanical performance of single PMUT is characterized using LDV in both air and water. Figure 10 presents an example of the LDV measurement results for uncoated PMUTs in both media, showing that PMUTs with larger membrane diameters exhibit lower-resonance frequencies and higher displacements. This behavior is due to the decreased overall stiffness of the larger membrane. Additionally, the resonance frequencies in water for each PMUT diameter are significantly lower than those in air, which is caused by the added loading effect of the surrounding water, as described by Equation (5).

### 4.1. Impact of Encapsulation on Single PMUTs in Air

**Figure 11:**
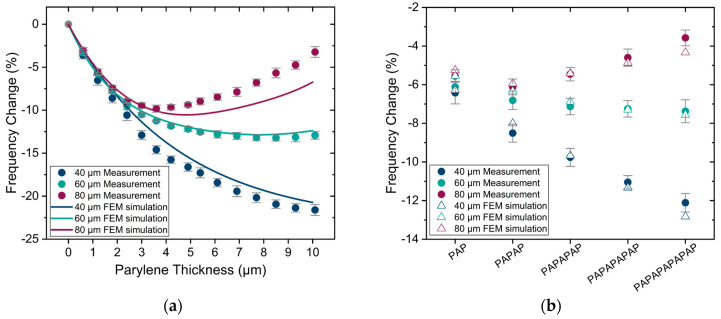
Measurement and simulation results of resonance frequency change in air (**a**) for different thicknesses of Parylene and (**b**) for encapsulation stacks. Error bars: final accumulated measurement error (1 sigma).

Figure 11 presents the measurement and the simulation results of resonance frequency change for uncoated and encapsulated PMUTs in air. To effectively compare changes across different diameters, the relative change is calculated per sample, and for similar samples the mean value is determined. Error bars represent the final accumulated measurement error (1 sigma). As it is shown in Figure 11, the measurements and the COMSOL simulations both demonstrate non-monotonic behavior for resonance frequencies, especially by increasing the thickness of Parylene, which matches with the trend observed in the analytical model shown in Figure 7. However, whereas the analytical model predicts the same percentage change for all diameters, the experimentally observed percentage changes for different diameters are not identical, which is likely due to deviations from the theoretical thin plate model [57]. Nevertheless, these frequency changes are very well reproduced with the FEM simulations for both Parylene and multi-stack encapsulation.

**Figure 12:**
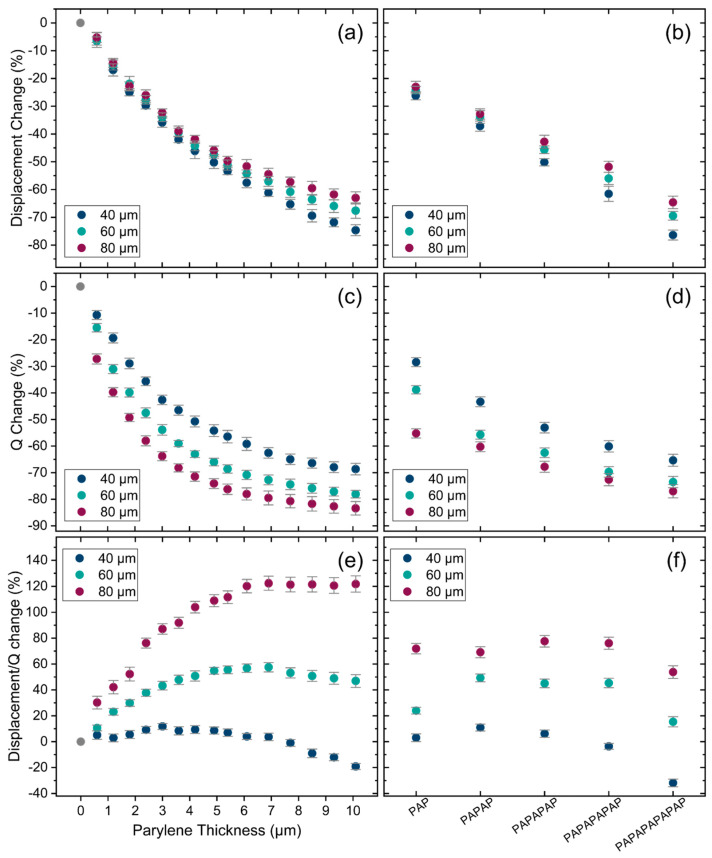
(**a**) Measured displacement change in air for different thicknesses of Parylene and (**b**) for encapsulation stacks. (**c**) Q factor change in air for different thicknesses of Parylene and (**d**) for encapsulation stacks. (**e**) Change in transduction efficiency in air for different thicknesses of Parylene and (**f**) for encapsulation stacks. Error bars: final accumulated measurement error (1 sigma).

Figure 12a,b illustrates the relative change in displacement, as experimentally measured using LDV, for different thicknesses of Parylene and for the encapsulation stacks. The displacement decreases with the increasing thickness of Parylene and the addition of Parylene/ALD layers in the encapsulation stacks. This change is mainly attributed to the increasing dissipation caused by increasing the encapsulation thickness. This trend is further confirmed in Figure 12c,d, which shows the relative change in Q factor derived from the LDV measurements. The Q factor also decreases with the increasing thickness of Parylene and encapsulation stacks. However, while the Q factor decreases comparably to displacement in terms of percentage, the rate of change differs. This can be seen in Figure 12e,f, which presents the change in transduction efficiency (displacement over Q factor) for varying thicknesses of Parylene and encapsulation stacks, respectively. The trend of the change in the measured transduction efficiency with Parylene thickness results is consistent with that of the analytical transduction factor presented in Figure 8. However, it differs in magnitude by a factor of 3. This discrepancy is most likely due to internal residual stresses in the membrane layers and encapsulations, which are not accounted for in the analytical model, as well as simplifications within the model itself.

Furthermore, also here a dependency on the PMUT diameter is observed, which is not expected in the analytical calculation. As with the case of frequency dependence of encapsulated devices, we believe that the deviation from the thin plate model, and potentially any error introduced by the presence of internal stresses, introduce a diameter dependence of the two quantities in question.

### 4.2. Impact of Encapsulation on Single PMUTs in Water

**Figure 13:**
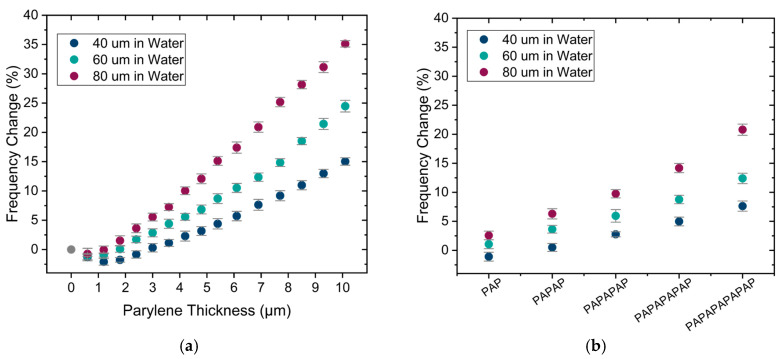
Measurement of PMUT resonance frequency change in water (**a**) for different thicknesses of Parylene and (**b**) for encapsulation stacks. Error bars: final accumulated measurement error (1 sigma).

Results of resonance frequency changes in water are shown in Figure 13 for encapsulations of Parylene and stacks. The resonance frequency is increasing by increasing the thickness of Parylene with a slight drop for the thinner parylene layers.

**Figure 14:**
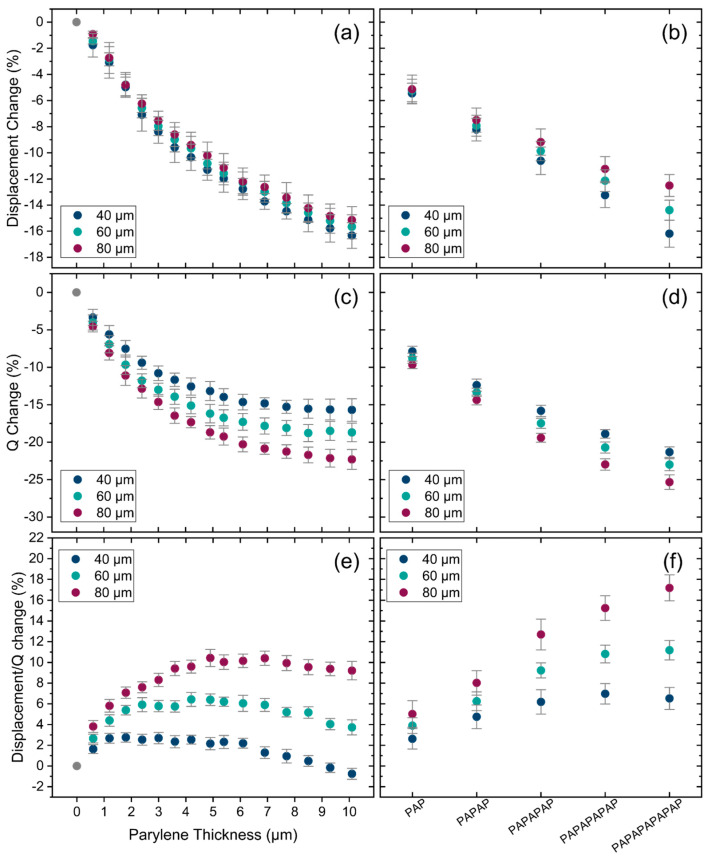
(**a**) Measured PMUT displacement change in water for different thicknesses of Parylene and (**b**) for encapsulation stacks. (**c**) Q factor change in water for different thicknesses of Parylene and (**d**) for encapsulation stacks. (**e**) Change in transduction efficiency in water for different thicknesses of Parylene and (**f**) for encapsulation stacks. Error bars: final accumulated measurement error (1 sigma).

The change in displacement in water due to the addition of encapsulations is presented in Figure 14a,b. Similar to air, the displacement in water decreases as the thickness and number of layers increase, although the reduction in displacement is much less in water. This is because PMUTs in water already possess a relatively low quality factor, and the added dissipation due to encapsulation is much smaller in the case of water than it is for the case of air, as illustrated in Figure 14c,d. This is a very important observation since this means that adding Parylene/ALD encapsulation to PMUTs, being part of an implanted device, will cause only a limited change in PMUT performance. Finally, the transduction efficiency results, shown in Figure 14e,f, exhibit a similar trend to the one observed in air and expected from the analytical calculations, again with an offset in the magnitude.

### 4.3. Impact of Water Mass Loading on Resonance Frequency

**Figure 15:**
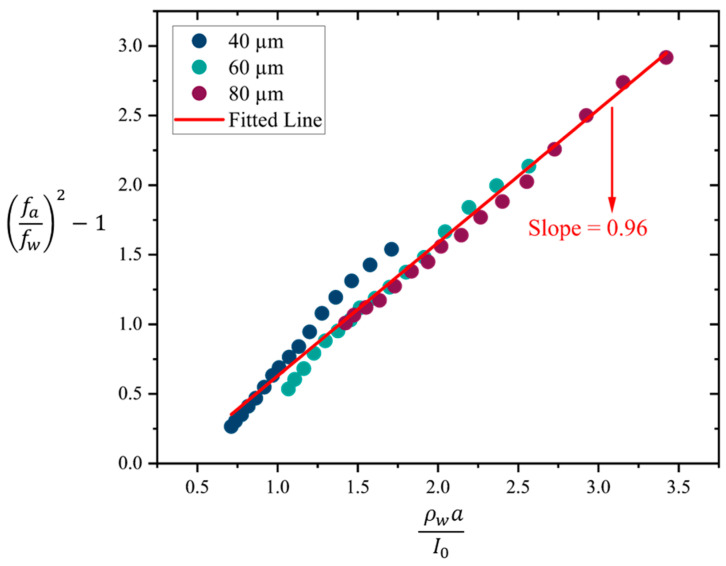
Plot to extract the value of the NAVMI parameter for PMUTs.

Here, we aim to calculate the NAVMI factor (Γ) using the measured resonance frequencies in air and water. As outlined in Section 3.2, Γ can be determined by the plotting of ${\left(\frac{f_{a}}{f_{w}}\right)}^{2}-1$ versus $\frac{\rho_{w}a}{I_{0}}$ as described in Equation (7), where the slope of the resulting plot corresponds to the value of Γ. Figure 15 presents this plot, derived from the resonance frequencies in air and water for the single PMUT devices of different cavity diameters and encapsulation layers. The red line in the plot represents the fitted line for all data points, with a slope of 0.96, which corresponds to the value of Γ. To the author’s best knowledge, this is the first of such empirical determination of the NAVMI parameter for MUT devices.

### 4.4. Acoustic Transmisson and Sensitivity of PMUT Arrays

Next to the evaluation of encapsulations on individual PMUT devices, the encapsulation of PMUT arrays is also studied. As described before, three types of arrays are tested, consisting of PMUTs with an individual diameter of 40 µm, 60 µm, and 80 µm.

**Figure 16:**
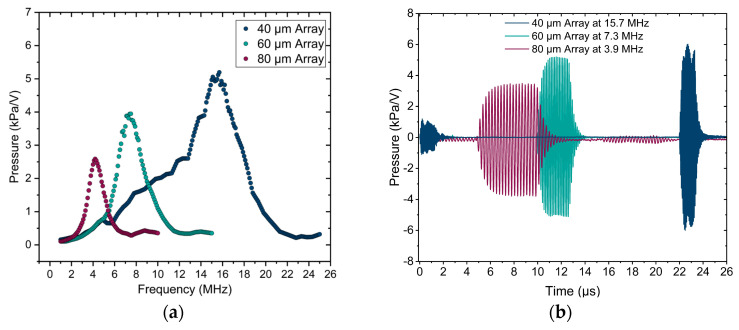
Acoustic response of PMUT arrays. (**a**) Frequency responses of uncoated arrays in the far field, showing peak frequencies of 15.7 MHz, 7.3 MHz, and 3.9 MHz for 40 µm, 60 µm, and 80 µm arrays, respectively. (**b**) Received pressures at the focal point for each array functioning at its peak frequency, converted from the received voltage using the sensitivity of the needle hydrophone.

In order to evaluate these PMUT arrays in their transmission mode, the pressure output was examined using the acoustic transmission setup in water as described in Section 2. Figure 16a presents the frequency responses of the uncoated arrays in their far field, with peak frequencies observed at 15.7 MHz, 7.3 MHz, and 3.9 MHz for the 40 µm, 60 µm, and 80 µm arrays, respectively. Each array was actuated at its respective peak frequency, and the focal point was determined by 3D scanning. The focal points for the 40 µm, 60 µm, and 80 µm arrays were located at 32 mm, 15 mm, and 8.4 mm, respectively. The pressure output for each array was recorded at their focal point. Figure 16b illustrates the pressure output of each array at the focal point and peak frequency, which were derived from the hydrophone signal using the sensitivity characteristics of the needle hydrophone. The pressure outputs of uncoated arrays at the focal point and peak frequency are 6.12 kPa/V, 5.17 kPa/V, and 3.44 kPa/V, respectively, for the 40 µm, 60 µm, and 80 µm arrays.

**Figure 17:**
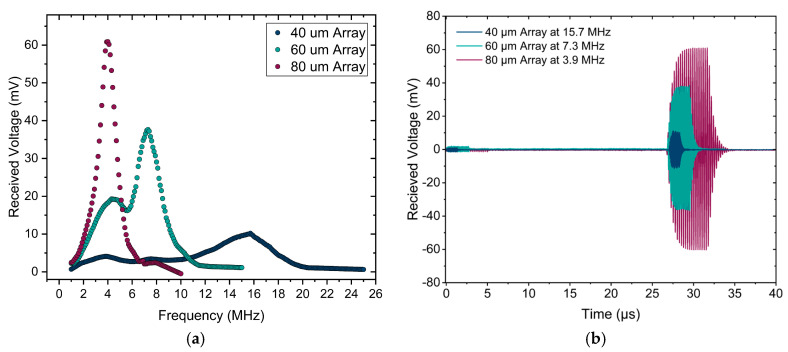
(**a**) Received voltage by the PMUT array during a frequency sweep of the acoustic signal delivered by the Olympus transducer, located at 4 cm from the array. (**b**) Received voltage signals for each array at their respective peak frequencies.

The encapsulation of PMUT arrays is also investigated, in case the arrays are used as receivers, using the method explained in Section 2. Figure 17a illustrates the received voltage during a frequency sweep at the distance of 4 cm from the Olympus transducer functioning as a pressure source. As expected, the results show the same peak frequency for each array, independent of the fact that the array is used as a transmitter or receiver. The received voltage signals recorded for each array at their respective peak frequencies are shown in Figure 17b. The received signal for 40 µm arrays is significantly weaker compared to the 80 µm array. This is because the beam width of the Olympus transducer becomes smaller as the frequency increases. The diameter of the pressure field of the transducer at 4 cm (the distance between the transducer and the array) is measured to be approximately 5.5 mm, 2.1 mm, and 1 mm for frequencies of 3.9 MHz, 7.3 MHz, and 15.7 MHz, respectively. For the 40 µm array, which operates at 15.7 MHz, the beam width is smaller than the size of the PMUT array (2.8 mm×2.8 mm), hence fewer PMUT elements are subjected to the pressure field. Consequently, the received voltage for the 40 µm array is clearly weaker compared to the 80 µm array, which is fully exposed to the acoustic beam. To determine the sensitivity, the received voltage for each array is normalized by the acoustic pressure of the Olympus transducer at the corresponding frequency. The obtained sensitivity for the 40 µm, 60 µm, and 80 µm arrays are 67.88 mV/MPa, 261 mV/MPa, and 691 mV/MPa, respectively.

**Figure 18:**
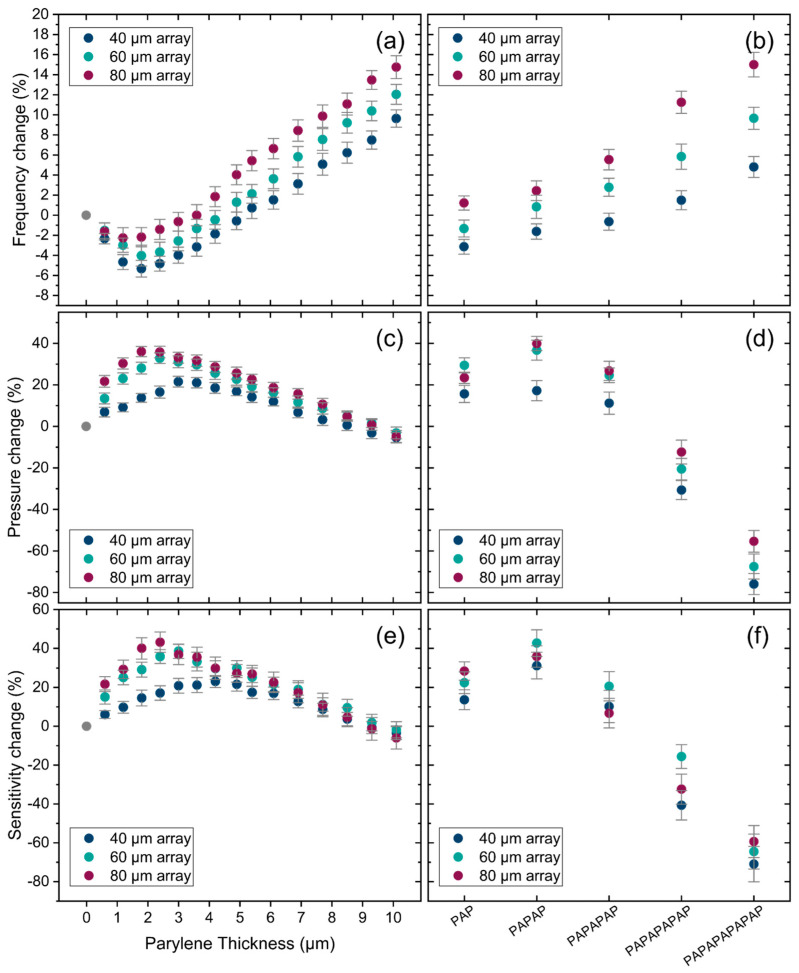
Transmission measurement results in water for PMUT arrays, (**a**) peak frequency change for different thicknesses of Parylene and (**b**) for encapsulation stacks, (**c**,**d**) pressure output change, and (**e**,**f**) sensitivity change for different thicknesses of Parylene and encapsulation stacks. Error bars: final accumulated measurement error (1 sigma).

After coating the PMUT arrays with encapsulation layers, their pressure output and sensitivity are again measured, and the percentage change in peak frequency, pressure output, and sensitivity for these encapsulated PMUTs is shown in Figure 18. The first set of plots shows the peak frequency change for different PMUT arrays as a function of Parylene thickness and encapsulation stacks. As expected, the peak frequency change for the PMUT arrays follows the same non-monotonic trend observed also for single PMUTs in the previous section.

Figure 18c,d illustrates the change in pressure output for different encapsulations, highlighting the significant impact of the encapsulation on PMUT performance. As the thickness of Parylene increases, the pressure initially rises for all PMUT arrays, reaching a maximum before decreasing. The optimal Parylene thickness varies among the arrays, with the 40 µm array achieving a 21% increase at 3 µm Parylene thickness, the 60 µm array showing a 32% improvement at 2.4 µm Parylene thickness, and the 80 µm array exhibiting a 36% enhancement at 1.8 µm Parylene thickness. These results indicate the potential of Parylene as an encapsulation layer in enhancing the acoustic performance of PMUT arrays. On the other hand, for encapsulation stacks, the pressure output improves for all three primary stacks, with the highest pressure output observed for PAPAP. However, for the thicker encapsulation stacks, the pressure output decreases significantly for all three arrays, suggesting that these thicker stacks are unsuitable for the encapsulation of the tested PMUT arrays. As shown in Figure 18e,f, the sensitivity changes closely following the pressure change results across different encapsulations.

The pressure output of arrays change non-monotonically by increasing the thickness of Parylene, while the membrane displacement measured for single PMUTs decreases consistently. Additionally, for encapsulation stacks—especially the thickest stack—the pressure output of arrays drops significantly (~70%), whereas the displacement of single PMUTs decreases only by ~15%. These complex behaviors of arrays with different encapsulations are likely due to changes in inter-element coupling, which are influenced by the encapsulation. Moreover, the shift in the peak frequency of the arrays due to the encapsulation will alter the interference pattern of the acoustic waves generated by the individual PMUTs in the array. The peak frequency, which corresponds to the acoustic wavelength (λ), changes while the PMUT pitch size (PMUT element spacing) obviously remains constant since it is part of the array design. Constructive interference occurs when the pitch size (p) equals λ/2. Under this condition of constructive interference, the directivity of the generated acoustic wave improves, minimizing sidelobes and concentrating acoustic pressure at the focal point.

**Figure 19:**
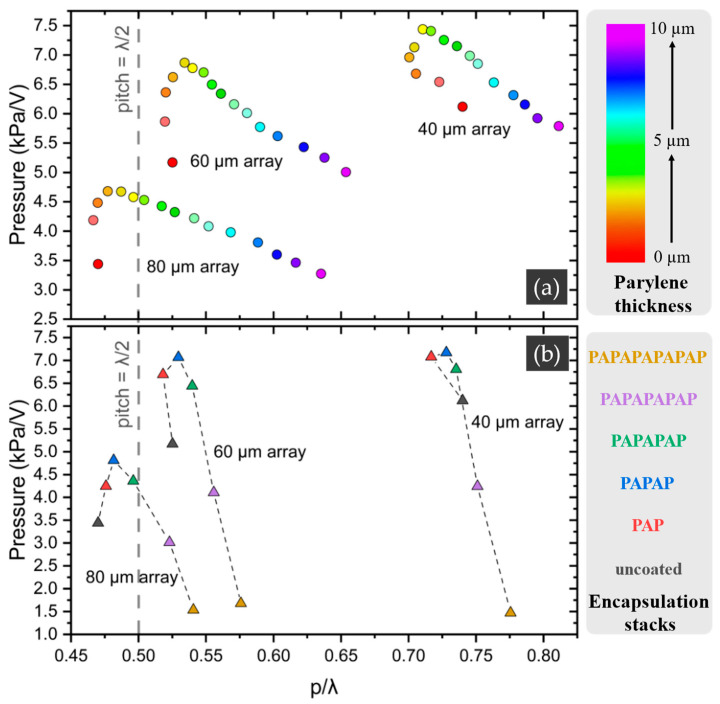
Pressure output of PMUT arrays as a function of p/λ for (**a**) different Parylene thicknesses and (**b**) different encapsulation stacks.

Figure 19 presents the pressure output of the arrays for different Parylene thicknesses and encapsulation stacks as a function of p/λ, where p is 70 µm for the 40 µm array, 122 µm for the 60 µm array, and 200 µm for the 80 µm array. As illustrated in the graphs, for both the different Parylene thicknesses and the different encapsulation stacks, there is a clear relationship between pressure output and p/λ. For the 80 µm and 60 µm array, the pressure output is maximized when the p/λ value is close to 0.5. For the 40 µm arrays, the highest pressure output observed at encapsulation results in a p/λ value which is closer to 0.70. This suggests the involvement of an additional mechanism, most likely the influence of encapsulation on inter-element coupling, which seems to be more pronounced for the 40 µm array.

## 5. Conclusions

This study presents a comprehensive evaluation of flexible encapsulation layers on piezoelectric micromachined ultrasonic transducers (PMUTs), aiming to make them suitable for implantable biomedical applications by providing the PMUTs with a biocompatible and hermetic enclosure. The effects of Parylene F-VT4 and triple ALD stacks, applied in various thicknesses and multilayer combinations, were investigated to assess their influence on PMUT performance. Laser Doppler vibrometry measurements in air and water for single PMUTs, alongside acoustic pressure output and sensitivity measurements in water, were conducted to analyze the impact on membrane dynamics and acoustic output efficiency.

The findings reveal a non-monotonic relationship between encapsulation layer thickness and resonance frequency in both air and water, driven by a balance between added mass and increased stiffness. For smaller encapsulation thickness, the resonance frequency is lowered due to the dominant effect of added mass. However, when the thickness increases, a critical thickness will be reached for which the influence of enhanced effective stiffness gains importance over the mass-loading effect, resulting in a subsequent increase in resonance frequency. This critical thickness differs for various sizes of PMUTs, as well as the actuation medium (air or water). Additionally, the membrane displacement of single PMUTs decreased with increasing encapsulation thickness, reflecting the influence of dissipation and enhanced stiffness. Although the decrease in displacement with increasing thickness occurred in both air and water, the decrease in water was significantly less than in air. Furthermore, we measured the NAVMI factor, which is, to the best of our knowledge, the first time that such a measurement was reported for MUTs. The obtained NAVMI factor provides valuable insight into the interaction between MUTs and their surrounding medium, allowing for more accurate estimation of resonance frequencies in different environments.

While the displacement of individual elements in water slightly decreased with increasing thickness, the acoustic performance of PMUT arrays in water is notably improved. Both the transmission and reception performance of arrays, which are the key parameters in the use of PMUT arrays for biomedical applications, are improved, with a peak at a certain thickness for each array (between 2 and 5 µm for only Parylene F-VT4). The improved non-monotonic acoustic performance of arrays could be due to changes in inter-element coupling and wave interactions. Our results suggest that specific encapsulation strategies, such as carefully tuned Parylene thickness or an optimized Parylene F-VT4/ALD multilayer stack, can even result in an improved acoustic performance.

In conclusion, the results support the use of Parylene F-VT4 and ALD stacks as viable, biocompatible, and hermetic encapsulation materials for PMUTs in biomedical applications, where precision and reliability are critical. Our results indicate that the encapsulation layers not only provide a hermetic protection of the PMUT arrays, but equally enhance their performance if the stack parameters are well-chosen. While the results presented in this work underline the impact of encapsulation on the dynamics and acoustic properties of PMUT arrays, or potentially any MUT devices, in an environment consisting of water, to evaluate the impact of encapsulation on MUT devices as part of a human implant, future tests are still essential, using saline instead of water as medium. Furthermore, in vivo tests should be performed to evaluate the influence of fibrous tissue growth around the device during the foreign body reaction (FBR). Fibrous tissue growth during the FBR is strongly dependent on many factors, such as the size and shape of the total implanted device, but also the surgical conditions, the implant location in the body, etc. Hence, such a test can only meaningfully be performed for a specific implantable device for a well-defined medical application.

## Figures and Tables

**Table 1:** Mechanical properties of materials used for calculation.

Material	Density (kg·m^−3^)	Young’s Modulus (GPa)	Poisson’s Ratio	References
Al	2710	69	0.33	[49]
AlS_9.5_N	3234	210	0.26	[50]
SiN	3200	167	0.23	[51]
Parylene F-VT4	1652	3	0.35	[52]
ALD Al_2_O_3_	~3100	171	0.3	[53]
ALD HfO_2_	~9800	166	0.3	[54]

## Data Availability

Data are contained within the article.
